# An interdisciplinary fetal neonatal neurology collaborative promotes integrative life-course brain health

**DOI:** 10.3389/fneur.2025.1725289

**Published:** 2026-01-07

**Authors:** Mark S. Scher, Harris Eyre, Steven Donn, James M. Roberts, Michael E. Msall, Carolyn M. Salafia, Richard Towbin, Peter Robinson, Ken Loparo, Michael Berk, Elena Moro, Valerie Smith, Susan Ludington, Nadia Badawi, Rod W. Hunt, Alistair Gunn, Harvey B. Sarnat, Kirthana Kunikullaya, Betsy Pilon

**Affiliations:** 1 Departments of Pediatrics and Neurology, Case Western Reserve University School of Medicine, Cleveland, OH, United States; 2 Brain Capital Alliance, San Francisco, CA, United States; 3 Center for Health and Biosciences, The Baker Institute for Public Policy, Rice University, Houston, TX, United States; 4 Meadows Mental Health Policy Institute, Dallas, TX, United States; 5 Institute for Mental and Physical Health and Clinical Translation (IMPACT), Deakin University and Barwon Health, Geelong, VIC, Australia; 6 Department of Psychiatry and Behavioral Sciences, Baylor College of Medicine, Houston, TX, United States; 7 Department of Pediatrics University of Michigan School of Medicine, Division of Neonatal-Perinatal Medicine, C.S. Mott Children’s Hospital, Michigan Medicine, Ann Arbor, MI, United States; 8 Obstetric Gynecology and Reproductive Sciences, Epidemiology and Clinical and Translational Research, Magee-Women’s Research Institute, University of Pittsburgh, Pittsburgh, PA, United States; 9 Department of Pediatrics, Section of Developmental and Behavioral Pediatrics, University of Chicago School of Medicine, Chicago, IL, United States; 10 Placental Analytics LLC, New Rochelle, NY, United States; 11 Institute for Basic Research, Staten Island, NY, United States; 12 New York Presbyterian, Brooklyn Methodist Hospital, Brooklyn, NY, United States; 13 Department of Radiology and Child Health, Mayo Clinic, Phoenix Children’s Hospital, University of Arizona College of Medicine-Phoenix, Phoenix, AZ, United States; 14 Berlin Institute of Health at Charité, Universitätsmedizin Berlin, Berlin, Germany; 15 The Jackson Laboratory for Genomic Medicine, Farmington, CT, United States; 16 Institute for Smart, Secure and Connected Systems (ISSACS), Case Western Reserve University, Cleveland, OH, United States; 17 Department of Electrical, Computer, and Systems Engineering, Case Western Reserve University, Cleveland, OH, United States; 18 School of Medicine, Deakin University, IMPACT, The Institute for Mental and Physical Health and Clinical Translation, Geelong, VIC, Australia; 19 Orygen, Parkville, VIC, Australia; 20 Florey Institute for Neuroscience and Mental Health, University of Melbourne, Parkville, VIC, Australia; 21 Department of Psychiatry, Royal Melbourne Hospital, Parkville, University of Melbourne, Parkville, VIC, Australia; 22 Centre for Youth Mental Health, The University of Melbourne, Parkville, VIC, Australia; 23 Grenoble Alpes University, CHU of Grenoble, Division of Neurology, Grenoble Institute of Neurosciences, Grenoble, France; 24 Midwifery, University College Dublin School of Nursing, Midwifery and Health Systems, Dublin, Ireland; 25 FP Bolton School of Nursing, Case Western Reserve University, Cleveland, OH, United States; 26 Cerebral Palsy Alliance Chair of Cerebral Palsy Research, The University of Sydney Medical Director and Co-Head of Grace Centre for Newborn Intensive Care, The Children’s Hospital Westmead, Sydney, NSW, Australia; 27 Department of Pediatrics for Rod Hunt, Monash University, Clayton, VIC, Australia; 28 Department of Physiology, Starship Children’s Hospital, University of Auckland, Auckland, New Zealand; 29 Department of Pathology, (Neuropathology) and Clinical Neurosciences, Cumming School of Medicine, Alberta Children’s Hospital Research Institute (Owerko Centre), University of Calgary, Calgary, AB, Canada; 30 Department of Physiology, Sri Chamundeshwari Medical College, Hospital and Research Institute, Bangalore, India; 31 Parent Advocate and Executive Director of Hope for HIE, West Bloomfield, MI, United States

**Keywords:** life-course brain health, transdisciplinary care, fetal-neonatal neurology, neural exposome, intersectionality

## Abstract

A proposed interdisciplinary fetal neonatal neurology collaborative offers life-course brain health training across three time-sensitive teaching opportunities. The educational organization includes a broad representation of inter-related fields. Formal training will re-enforce career-long learning that fosters creative thinking. Acquiring a life-course perspective of brain health can contribute solutions to the global public health crisis involving neurological and mental health disorders across the lifespan. Teaching transdisciplinary interventions begins with parental childhood and reproductive health which will influence the maternal-placental-fetal triad throughout pregnancy into labor and delivery. The second teaching opportunity focuses on the symptomatic minority who receive neonatal neurocritical care and convalescent care. The third educational cluster focuses on improving clinical skills as the unrecognized majority of children present over the preschool years with continued development through the school years. Teaching preventive neurology and mental health introduce proactive interventions that more effectively support rescue and reparative choices into adulthood. The science of uncertainty will be taught to all stakeholders that integrates information to improve critical thinking skills. This tripartite interdisciplinary educational program will help trainees distinguish adverse effects from neurodegeneration on primary fetal neuroplasticity mechanisms from secondary pathways based on systems-science. Supervised clinical experiences during each rotation will supplement didactic teaching with input from each trainee’s mentoring committee. Future providers will learn to anticipate adaptive from maladaptive disease pathways to prepare for career-long experiences. Curriculum topics will focus on brain health strategies that differentiate resilience from vulnerability based on time-dependent gene–environment interactions. Attention to structural, social and environmental drivers of health will incorporate intersectionality perspectives into equitable neuroprotective plans. Training will engage, educate and empower women to improve brain health for themselves and their children. This interdisciplinary collaborative program will apply real-world situations to encourage research development that will narrow the knowledge-practice gap. Continuity of brain care bundles will enable providers, women, and their families to achieve brain health across each and successive generations. A lower global burden of neurologic and mental health disorders will contribute to an improved quality of life with greater economic prosperity.

## Concepts and structure of a proposed interdisciplinary fetal neonatal neurology collaborative

This proposed interdisciplinary fetal neonatal neurology collaborative (IFNNC) is intended to promote global brain health practices across the lifespan. Career-long learning experiences will benefit from formal fetal-neonatal neurology training. This multi-authored description of this training program introduces new information of educational details that complement details in an earlier companion publication by the corresponding author (1). Connecting knowledge silos among multiple inter-related disciplines will more effectively address the worldwide public health crisis confronting persons with neurologic and mental health disorders (2). Interplay between women’s and children’s healthcare constitute the core organizational structure for this IFNNC. Time-dependent neuroprotective intervention recommendations will be taught across three time-sensitive developmental periods starting during reproductive life, throughout pregnancy into neonatal life followed by the preschool and school years. This educational structure offers the trainee a life-course perspective of brain health (3, 4). Prioritizing intersectionality will improve critical thinking when offering clinical decisions, merging sex, gender, cultural, ethnic and economic factors (5, 6) to achieve equitable brain health delivery. Biological sex will identify important genetic and hormonal factors based on parents’ reproductive and pregnancy healthcare histories. Gender identity will represent a multidimensional construct that is shaped by worldwide social and cultural diversity that influence healthcare across successive generations.

**Figure 1:**
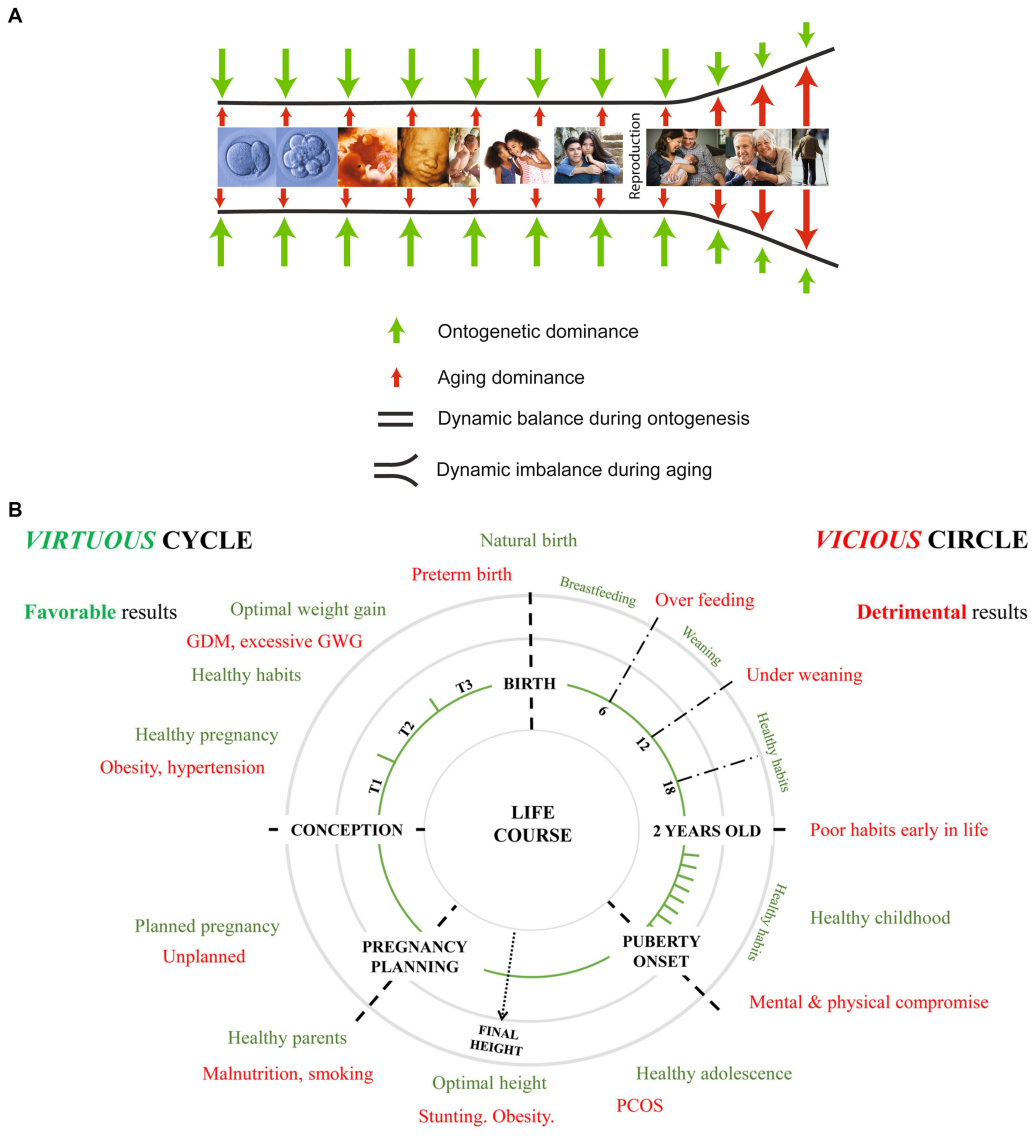
**(A)** A pictorial representation of the human life cycle depicts the dynamic interactions that connect developmental and aging processes. Representative images begin with the fertilized ovum and blastocyst, continuing through embryonic and fetal development. Following birth, images of the life cycle of women and their partners follow developmental and aging time periods are offered that will be repeated across successive generations [modified from (12)]. **(B)** Healthy (green color) women’s health practices help reduce unhealthy (red color) health choices over women’s life cycle for themselves and their children. Parent’s childhood health influences reproductive, pregnancy, childhood and adult health with intergenerational effects. Endogenous and exogenous toxic stressor interplay through gene–environment interactions is tracked across each life cycle with adaptive and maladaptive neuroplasticity expressions. GDM, gestational diabetes; GWS, gestational weight gain: PCOS, polycystic ovarian syndrome; T1, first trimester; T2, second trimester; T3, third trimester [modified from (11)].

Women’s childhood health into puberty through menopause defines matrescence or motherhood during which planned or unplanned pregnancies will have life-course brain health effects (7). Childhood and preconception histories of women and their partners influence clinical decisions regarding maternal-placental-fetal triad health throughout pregnancy, labor and delivery. This educational approach considers primary fetal brain effects as well as secondary consequences based on complex system interactions. Resilience or vulnerability of the woman and her partner reflect gene–environment interactions (8) that influence their child’s dynamic neural exposome beginning with conception. Exposomic effects will be represented by phenotypic diversity expressed over the first 1,000 days (9) that later will guide developmental and aging effects (10). Understanding bidirectional relationships between women’s and children’s healthcare (11, 12) contributes to brain health strategies by learning to choose appropriate neuroprotective options across the lifespan (Figures 1A,B). Neurologic disorders often remain unrecognized based on current prenatal testing. Therefore, knowledge of developmental neurology will be stressed during IFNNC training that will help close this knowledge-practice gap. Anticipation of antepartum and peripartum disease pathways potentially will influence intervention choices for the maternal-placental-fetal triad. A minority of children with neonatal brain disorders will require resuscitation and neurocritical care. An unrecognized majority will alternatively present with age-dependent expressions of neurologic and mental health disorders during the preschool years.

Care bundles were first proposed in the United States by the Institute of Health Care Improvement to identify and combine at least three required interventions to evaluate outcomes.^1^ IFNNC practice, education and research activities will introduce neuroscience knowledge that fosters the development of brain care bundles designed to improve life-course brain health. Transdisciplinary clinical decisions by all stakeholders must first be learned to acquire abilities to apply the most effective interventions using these care bundles (13).

Current one-year training has been suggested for neonatal neurocritical care trainees in preparation for a certification examination, endorsed by the American-based United Council of Neurological Subspecialties organization.^2^ The proposed IFNNC will more effectively connect knowledge silos among multiple neuroscience-related disciplines when offered over a 2 year period of formal fetal-neonatal neurology training. This more extensive educational opportunity will be better able to teach neurologic and mental health care delivery as career-long experiences are later accrued. Fetal-neonatal neurology training evaluation metrics were previously discussed involving each collaborative rotation (1). Further details are offered in this review based on three interactive clinical-teaching venues (Table 1). Performance metrics will be based on educators’ descriptive summaries from multiple fields of study during each teaching rotation, combined with classroom performance to gage the trainee’s readiness to take the final certification examination.

**Table 1:** IFNNC training with serial evaluations*.

Time-dependent clinical venues	Trainee evaluation metrics^#^
Preconception time-period ◦ Parental pediatric health ◦ Obstetrical services for women and partners First trimester assignments of maternal levels of care ▪ Prenatal imaging ▪ Biomarkers Second trimester surveillance ◦ Maternal levels of care ▪ Anatomic surveys with additional testing ▪ MFM and neonatal consultations MFM interdiscinary service ◦ Fetal neurology consultations ◦ MFM conferences ◦ Brain sonographic and mri fetal neuroimaging correlations Third trimester surveillance with assigned diagnsotic testing Maternal hospitalizations Peripartum and neonatal services ◦ Labor and deliverywith resuscitation needs ◦ Acute followed by convalscent stages of care ◦ Discharge plannng Pediatric services ◦ Primary care referrals ◦ Intervention program services ◦ Pediatric subspecialty and therapy referrals ◦ Inpatient/outpatient referrals ▪ Emergency room ▪ Pediatric intensive care ▪ Epilepsy service ▪ Sleep service Continuity of partnerships with parents and advocacy groups	Evaluation of parent’s childhood/reproductive health care ◦ Planned versus unplanned pregnancy health status ◦ Communicable/non-communicable diseases ◦ Adolescent pregnancies ◦ Structural, social, environmental health drivers in LMIC or hic healthcare deserts First educator cluster evaluations ◦ First trimester placental implantation/cord abnomalities ◦ Interpretation of aneuploidy, WES, GWAS results ◦ Tests for trimester-specific maternal communuicable/non-communicable diseases ◦ Distinguish brain anomalous from destructive lesions ◦ Consider brain effects from systemic maternal-placental-fetal triad illnesses ◦ Serial assessments of fetal brain structure/function disease pathways ◦ Outpatient clinic and MFM conferences ◦ Maternal hospitalizations including ICU care ◦ Peripartum monitoring modalities through labor and delivery Second educator cluster evaluations ◦ Resuscitative /early ICU stabilization with acute neonatal interventions ◦ Subacute-convalscent interventions ◦ Organ-system specific conditions requiring pediatric subspecialty services ◦ Nursing interventions-therapy services ◦ Individualized developmental infant intervention approachs Third educator cluster- evaluations ◦ Health maintenance/wellness programs after discharge ◦ Early intervention program referrals ◦ The medically fragile child ◦ Neuropalliative care ◦ System-specific surveillance throughout childhood ◦ Acute disease presentations requiring intervention adjustments ◦ Collaborative experiences during adult age transitions Transdisciplinary care assessments ◦ Demonstrate critical thinking for diagnoses and interventions ◦ Qualitative evaluations of communication skills among stakeholders ◦ Qualities of neurohumanism

*Adapted from reference 13. #Performance metrics of competence based on educators’ descriptive summaries within each teaching cluster compared to classroom test results help guide trainee readiness for the final certification examination.

Future IFNNC certification will require documentation of interdisciplinary clinical competence that stress cultural neuroscience concepts (14–17). Such efforts will contextualize behavioral, genetic, neural and physiological processes for real-world global practice applications. These perspectives will be applied to future curricular revisions, utilizing evidenced-based systematic reviews that guide educational strategies to improve outcomes (18). Shared decisions among all stakeholders in this collaborative will be taught to more effectively select among preventive, rescue and reparative neuroprotective intervention options which will be calibrated to each community’s available resources (8).

The broad range of IFNNC curriculum topics previously discussed (1) will collectively reduce the knowledge-practice gap that presently separates siloed disciplines. Cognizance of health or disease experiences during parents’ childhood through the reproductive years of women and their partners will strengthen medical decisions during pregnancy. The maternal-placental-fetal triad followed by neonatal and early childhood health establish foundational brain connectomes that rapidly mature before the child’s second year of life. Brain health subsequently will be maintained or compromised through childhood into adulthood, dependent on a person’s resilience or vulnerability (19–21). IFNNC partnerships with government, non-government, consumer, industry, and lay advocacy organizations will help advance practice, education and research efforts inclusive of resource-challenged communities (22). Critical thinking with agency (23, 24) will promote life course clinical decisions, combining forethought, implementation, self-management and learning through mindfulness and adaptation by stakeholders (25). Knowledge that integrates social, environmental, genetic, molecular, cellular, tissue and multisystem etiologic pathways (26) will better anticipate unrecognized neurologic and mental health disorders. This will compensate for prenatal and postnatal diagnostic limitations to detect phenotypic features. Population-generated data will be applied for person-specific brain healthcare decisions, reflecting unique dynamic neural exposome expressions during maturation through aging (9). More effective neuroprotective choices during critical/sensitive neuroplasticity periods contribute to life-course brain health. Subsequent clinical decisions apply retrospective reassessments as additional knowledge is acquired. Considerations of neuroplasticity processes must differentiate experience-independent, expectant and dependent pathways, particularly during critical-sensitive time periods (27) across the life cycle. Empowering women to participate in healthcare decisions will maintain their trust and engagement (7, 18) to sustain brain health for themselves and their children based on earlier clinical expressions of developmental neuroplasticity over the first 1,000 days. Prenatal, neonatal, and preschool brain health choices will prepare children later during their school years to support adult brain health experiences in the workplace into senescence.

Consideration of gene–environment interactions across each person’s lifecycle involve endogenous and exogenous factors that comprise the dynamic neural exposome (28). These will be expressed as positive or negative toxic stressor interplay (9). Adult brain health strategies will later adjust to brain and mental health diseases with aging. Applications of life-course (29) and developmental origins of health and disease (30) concepts will help improve the trainee to more accurately predict intragenerational and transgenerational outcomes. Adult neurologists and mental health providers will contribute to real-world IFNNC training into old age by introducing the trainee to a life-course brain health care perspective.

Neurohumanities education further enriches IFNNC training by integrating creative ideas within multi-media art space used by all stakeholders (31). Merging intellectual and emotional contributions promote shared clinical decisions that foster intersectionality based on neuroaesthetic principles (32). Artistic expressions sustain or restore brain health by valuing human sensibilities and empathy through memory and shared storytelling about a person’s life cycle experiences. These efforts will help strengthen transdisciplinary brain care decisions.

Worldwide collaborative efforts are already represented by the World Health Organization (WHO) (33), World Economic Forum (34), the Commonwealth Fund (35), the European Brain Council (5), the European Academy of Neurology (36) and the McKinsey Institute (34). These efforts collectively support the WHO 17 sustainable goals that propose to expand the breadth and depth of healthcare delivery across diverse communities and nations (37). Life-course brain health recommendations must integrate knowledge of preconception, pregnancy, birth and childhood experiences (38) to more optimally evaluate adult preformances (21) in the workspace (19). Recognition of structural, social and environmental drivers of brain health requires considerations across the life cycle. Advocacy for brain health equity must address those living in low to middle income countries and high-income country maternal healthcare deserts (39–44) by recognizing that intersectionality influences global health. Empowering women to participate in their own healthcare (45) will benefit children’s development and performance (46), improving adult quality of life and economic prosperity. The following three IFNNC clusters of provider-educators constitute synergistic efforts that contribute to teaching life-course brain health. Monitoring progress of trainees’ goals and objectives will be assisted by their respective mentoring committees.

### The preconception and fetal neurology IFNNC cluster

This first IFNNC cluster of provider-educators integrates knowledge of parental childhood health experiences with reproductive and pregnancy health care decisions. Pregnancy planning (47) more favorably will introduce preventive health care bundles (13). Physicians, nurses, midwives, and doulas collectively can better support prospective parents’ health decisions during pregnancy through delivery. Brain health bundles begin with wellness strategies that encompass nutrition, weight control, treatment of pre-existing medical and mental health conditions, and lifestyle choices to positively influence sleep, diet, exercise, stress-management and recreational substance choices. These activities begin during the girl’s childhood before ovulation and continue during her reproductive ages. Increasing attention to periconceptional events when planning pregnancy provide opportunities for interventions during this little studied component of pregnancy. This approach applies particularly to the use of assisted reproduction therapies during the crucial and sensitive time of periconceptional adaptation (48).

Opportunities for IFNNC consultations arise following pregnancy confirmation, guided by the recognition of risk factors or disease expressions. Obstetrical or maternal-fetal medicine referrals to fetal neurologists (49) are usually initiated based on prenatal genetic or neuroimaging results that implicate altered or impaired fetal brain development. Fetal neuroimaging advances that combine ultrasonographic and MRI data will help detect a greater range of brain malformations. Clinical insights based on integrative science however must also anticipate falsely negative or equivocal genetic or imaging test results. IFNNC training will strengthen fetal neurologists’ consultative insights based on participation in high-risk maternal-fetal-medicine conferences, prenatal consultations alongside neonatologists, and during hospitalizations of acutely ill women (1).

IFNNC training will benefit from working knowledge of diagnostic and therapeutic choices applicable during all levels of maternal care, ranging from non-pharmacologic wellness practices to complicated medical or surgical interventions (49). Worsening prenatal diseases and adversities necessitate reassessments that potentially impair the developing fetal brain. Infectious, hypertensive, endocrinologic and autoimmune disorders exemplify the broad range of maternal conditions that increase risks for fetal and/or neonatal brain lesions (9). Knowledge of maternal-placental-fetal triad surveillance tools offer opportunities to interprete women’s urine, serum and amniotic fluid test results that represent maternal, placental-trophoblastic and fetal-parental genetic biomarkers (1). Sonographic anthropometric and physiologic indices document fetal growth parameters combined with doppler indices and biophysical scores. Serial sonography compared with structural and functional MRI biomarkers help detect anomalous or destructive fetal brain lesions. Knowledge of neuroembryology and developmental neuropathological lesions enhance fetal neurology perspectives as unrecognized brain anomalies or injuries are considered. This neuroscience knowledge of prenatal brain connectivity will help compensate for sensitivity and specificity testing limitations that potentially offer falsely negative and positive results (50). Learning transdisciplinary decisions help stakeholders adjust to worsening health conditions throughout pregnancy. Maternal-fetal medicine and pediatric subspecialty consultations may be needed to evaluate more complicated conditions such as maternal infectious (51, 52), diabetic (53) and hypertensive disorders (54), fetal diagnoses such as intrauterine growth restriction (55), or organ-specific conditions such as congenital heart (56), diaphragmatic (57), and posterior fossa brain anomalies that require surgical considerations (58). Transfer to higher level medical centers offer women more complex fetal medical-surgical interventions that may require EXIT procedures during labor and birth.

Socio-demographic profiles, geographic location and resource availability currently determine intervention choices. User-friendly personal devices with artificial intelligence software will help empower women to share data with their providers before and during pregnancy that improve clinical decisions (59) such as monitoring for anemia (60, 61), glucose dysregualtion (59), hypertension (59) and mental health disorders (62) during low or high-risk pregnancies. Women unfortunately still receive inadequate prenatal healthcare secondary to poor health literacy, barriers to medical access or limited community resources. Educating them to apply real-world proactive preventive measures provide early neuroprotective intervention choices that will narrow this healthcare gap as delivery of their child approaches.

### The peripartum and neonatal neurocritical care IFFNC cluster

Antepartum health care by the first IFNNC provider cluster facilitates peripartum and neonatal clinical decisions by those participating in the second teaching cluster. Peripartum MPF triad surveillance includes monitoring of fetal heart rate patterns, uterine pressure, oximetry and fetal scalp blood pH^1^. Women’s medical status throughout labor and birth may deteriorate associated with different clinical scenarios such as clinical chorioamnionitis [i.e., “the triple I response” (63)], isolated or recurrent fevers (64, 65), unanticipated hemorrhagic events (66) or acute maternal cardiopulmonary decompensation (67). This brief time period may be the first opportunity to consider the loss of fetal well-being broadly defined as fetal distress (68). Present biomarkers however do not reliably predict the timing or progression of fetal brain injuries (69, 70). Providers can only anticipate adverse events that potentially represent disease pathways contributing to brain lesions. Sentinel events are comparatively less often encountered, requiring more rapid interventions with greater risks for brain injuries. Abruptio placenta, uterine rupture, cord prolapse, or acute maternal physiologic decompensation consequently require rapid delivery options, including instrument-assisted births or cesarean section.

The trainee’s understanding of the biologically robust peripheral chemoreflex will be essential. This protective response is activated with intrapartum fetal hypoxic events, usually identified with vagal responses during active labor. Physiologic hypoxia more often will be expressed, associated with natural protection to preferentially fetal brain, heart and adrenal gland. This reflex is particularly important for these vital organs during transient periods of anaerobic metabolism while reduced oxygen concentrations are delivered to less vital fetal organ systems (71). Working knowledge of fetal brain responses during more difficult prenatal-to-neonatal transitions help anticipate maladaptive physiologic responses from pathologic hypoxia which requires alternative resuscitative care paths. Gross placental, cord and uterine abnormalities observed after delivery may alert providers to possible antepartum diseases before during parturition, such as morbidly-adherent placental tissues, discoloration, calcifications, infarctions as well as cord coiling, knots, and marginal-velamentous insertions (72). These gross descriptions will require histopathologic confirmation before considering outcome correlations. Perinatal pathologists will offer retrospective interpretations based on a broad range of acute or chronic descriptions, as exemplified by the fetal inflammatory response, ischemic placental syndrome, maternal immune activation or villous dysmaturity (1). These pathological biomarkers individually or collectively may have negatively impacted antepartum maternal-placental-fetal triad health with associated fetal brain injuries. These conditions later adversely influence outcomes during a problematic labor and delivery. Future identification of perinatal pathological findings using novel antepartum tests such as holistic MRI of the mother, placenta and fetus (73, 74) will contribute to more effective peripartum obstetrical interventions for the next generation of providers.

Neonatal clinical signs immediately following delivery more often represent transient disorders of consciousness, represented by improving Apgar scores that reflect resumption of multi-systemic homeostasis. Progression to neonatal encephalopathy with or without seizures less commonly occurs and more likely require staged neuroresuscitative interventions, including therapeutic hypothermia and antiepileptic medications. Gestational-age specific intervention choices will be influenced by conditions of prematurity for this larger population of neonates who usually require longer periods of intensive care. Suspicion of cerebrovascular occlusive or hemorrhagic events as well as craniocerebral trauma may necessitate immediate neuroimaging. Diagnostic analyses taught to trainees must broadly consider acute as well as chronic timing of diverse etiopathogenetic pathways based on prospective interpretations of available clinical and laboratory information. Learning disease descriptors using Apgar, Sarnat (or similar systems), followed by Thompson and Hammersmith scores integrate anthropometric measurements, clinical signs of dysautonomia, and non-neurologic examination findings categorize clinical disease progression or resolution before and after 24 h of life. Serial blood gasses, cellular blood counts and organ-specific biochemistry results contribute to these analyses (75). Multi-systemic abnormalities may contribute to secondary neonatal brain disorders, exemplified by cardiopulmonary, inflammatory, hematologic or inherited metabolic-genetic disorders (76). Appreciation of multiple assessments guide diagnostic and prognostic decisions throughout neonatal hospitalization.

Neonatal clinical mimicry represents diagnostic challenges for the trainee given that impaired levels of consciousness represent genetic and/or acquired disease states associated with diverse timing, etiologies and outcomes. Genetic vulnerabilities for example exacerbate acquired disease effects from sedative or anti-epileptic medications, the fetal inflammatory response syndrome, neonatal abstinence states or even the use of therapeutic hypothermia (9). Serial neonatal neuroimaging, electroencephalography, placental-cord histopathologic findings (77) and even neuropathologic considerations after fetal or neonatal demise (78) may collectively or individually offer crucial diagnostic findings. Interpretative knowledge by trainees of serial assessments strengthen real-time diagnostic assumptions regarding timing and etiologies of disease pathways. Interdisciplinary collaborations will help improve recognition of complex disease pathways that contribute to the expression of neonatal neurologic disorders (79).

Postnatal microarray, exome and high-throughput genome testing help identify specific genetic biomarkers (80) associated with pathologic correlations. Complex antepartum gene–environment interactions may have already impaired maternal-placental-fetal triad health with preconception health risks despite no pathologic genetic test results. Abnormal phenotypic signs may also represent acquired fetal or neonatal neurologic disease states influenced by variants of uncertain significance rather than represented by definitive pathologic genetic variants. Genetic vulnerabilities also lower fetal physiologic defenses which otherwise might have avoided or minimized acquired injuries during antepartum or intrapartum stresses.

Neonatal neurocritical care education integrates acute, step-down and convalescent interventions as multi-systemic diseases contribute to potential neonatal brain injuries. Neuroprotective choices will be guided by the child’s gestational maturity as a preterm or full-term neonate which are associated with specific disease pathways. Adjustments in care are required to learn as new or worsening clinical repertoire emerges. Varying severities of neonatal encephalopathy, seizures and cerebrovascular events (26) involve diverse clinical presentations representing communicable and noncommunicable diseases. Serial brain care bundles require interventions that are person-specific to initially achieve short-term benefits (13). A broad range of maternal-placental-fetal triad and neonatal diseases can be associated with identified pathogens, multisystemic disease states, and xenobiotic exposures from prescribed, environmental or recreational drugs. Less severe encephalopathies present diagnostic challenges given arousal, tone and localization-specific neurologic deficits that are more difficult to identify and interpret. These situations are exemplified by mild hypoxic–ischemic encephalopathy (81) based on modified Sarnat criteria or self-limited clinical or electrographic seizures without accompanying encephalopathic signs (82). These non-specific phenotypes represent diagnostic, therapeutic, and prognostic challenges for the trainee that depend on a child’s resilience or vulnerability throughout prenatal and neonatal life.

An IFNNC offers multifaceted interventions that more effectively can restore neonatal physiologic homeostasis by identifying and reducing prenatal and postnatal endogenous and exogenous toxic stressors (83, 84). Communication among pediatric subspecialists, nurses, therapists and parents help maintain trust and engagement as shared decisions are implemented. Environmental adjustments to medical interventions with parental participation help improve state regulation, achieve positional comfort for motor and cardiorespiratory functions, minimize pain and stress, and optimize feeding and behavioral interactions. These efforts constitute important non-pharmacologic developmental care interventions to be emphasized for the trainee (13). Newborn Individualized Developmental Care and Assessment Programs or related programs (85, 86) provide comprehensive approaches to assess neonatal development and behavior to better select person-specific interventions. Skin-to-skin contact and breastfeeding with family participation remain essential proactive brain care bundles despite resource challenges. Short-term improvements in brain function followed by long-term benefits reflect enhanced structural and functional neuronal connectivity that can be achieved even with low-impact interventions (87, 88). Developmental care interventions continue to protect infants following discharge even in low-middle income countries and high-income country healthcare deserts where resource limitations prevail (86).

### Neurological, developmental and behavioral pediatrics IFNNC cluster

Wellness programs by primary care providers offer continuity of brain care bundles for children and families, who contribute to the third IFNNC cluster of provider-educators following discharge. Understanding developmental neuroscience principles guide clinical decisions for the trainee during the child’s first years of life as neurologic and behavioral health practices are implemented (89). Early intervention programs utilize serial clinical biomarkers such as general movement and Bayley neurodevelopmental assessments to detect domain-specific delays to offer time-sensitive treatments that avoid or lessen permanent sequelae.

The first critical/sensitive period of neuroplasticity will be foundational for life-course brain health, given that 80% of brain connectivity have been established with segregation and specification of function during these first 2 years of life (90). Wellness practices focus on nutritional status, immunization schedules, developmental milestone tracking and multi-systemic health surveillance to minimize cumulative negative effects from prenatal and postnatal toxic stressor interplay (9). Developmental care practices with multi-modal therapies remain important to learn for the trainee during the preschool years, particularly for high-risk children with identified fetal or neonatal complications. Coordinated evaluations by primary care physicians, neonatologists, pediatric neurologists, developmental-behavioral pediatricians, mental health providers and other multi-specialty therapists provide comprehensive tracking of the child’s developmental progress, particularly for those who remain medically fragile children with higher risks for neurodevelopmental disorders. Learning effective communication skills between parents and providers through 3 years of corrected age accommodate for the developmental ages of preterm survivors.

Communicable or non-communicable diseases challenge children’s health throughout the first 5,000 days before school entry, necessitating subspecialty involvement during situation-specific outpatient or inpatient illnesses (91). Severely impaired children particularly those receiving neuropalliative care will more likely require emergency room or pediatric neurocritical care evaluations (92) with treatment re-adjustments including initiation or changes in neurorehabilitation (93). Adverse childhood experiences introduce structural, social and environmental barriers to brain health. Bio-social consequences based on these experiences must be overcome to improve child health care (94), particularly in vulnerable communities. These adverse experiences range from poverty, xenobiotic exposures, racial discrimination, trauma, abuse and neglect based on negative family dynamics and parent’s mental health disorders.

School preparation involves serial developmental screening and interventions that help families adjust to their child’s underlying learning and regulatory behaviors. Parental-physician questionnaires assist with early detection of developmental disorders. Standardized psychometric tests provide domain-specific language, cognition, motor, and social-adaptive assessments at older ages. Deviations from normative performances are recognized using age, gender, race and ethnicity-sensitive testing parameters to help design individual educational plans. Trainees must appreciate neuroscience principles (95) that help guide educators’ to meet each child’s scholastic needs. Functional neurodivergence (96) expressed by many children include those with attention deficit, learning and autistic spectrum disorders. Positive neuroadaptation (97) more likely can be achieved following earlier interventions as greater functional motor, communicative and neurodevelopmental abilities are expressed. More severely impaired children will require sustained special educational plans throughout their school years, calibrated to each person’s designed brain health-educational care bundles. Trainees must recognize that greater functionality can be achieved even for these more vulnerable children based on appropriate health and educational refinements (98).

Phenotypic disease classifications assist with appropriate interventions for children identified with specific motor, epileptic, autistic spectrum, intellectual, cognitive-behavioral, and other mental health disorders. Worldwide registries exemplified by the Cerebral Palsy Registry provide epidemiologic and interventional resources that help improve interdisciplinary diagnostic and research collaborations (99). Genetic testing identifies specific brain disorders such as those children with cerebral palsy, Down’s syndrome and Fragile X disorder who can then receive more focused interventions. Transcriptional abnormalities classified as variants of uncertain clinical significance re-enforce the need to offer the most effective interventions despite no identified pathologic genetic biomarkers. Future research will strengthen causal genotype–phenotype relationships by incorporating individual cases and smaller series into larger population data bases. These efforts will yield greater statistical significance by utilizing computational science with artificial intelligence applications through machine learning (100).

School readiness and performance rely on serial observations as each child’s neurodevelopmental and educational progress continue to be monitored. These assessments help those children with persistent scholastic difficulties. Trainees need to learn of IFNNC-generated pre-school developmental assessments. These strategies assist educators to provide children with the most beneficial individual educational plans to address neurobehavioral-cognitive disorders. A multidisciplinary school team coordinates interventions with families to offer recommendations extended at home outside of school times. Neurologic, developmental-behavioral, neurorehabilitative and child psychiatric specialists offer information that help design neuroscience-based educational plans to meet each child’s neurologic and mental health challenges. All students benefit from a balance between individualized educational attention while supporting learning within a mainstreamed classroom environment for children with all levels of ability (101). A major challenge remains in resource-limited communities where educational as well as healthcare barriers prevail (102). Diagnostic Statistical Manual-version 5 diagnostic criteria help identify and track mental health disorders for children who also experience scholastic challenges, such as attention deficit hyperactivity, autistic spectrum, and cognitive delay with anxiety, mood and thought disorders (103). Anxiety and mood disorders for example can significantly impact educational goals, requiring adjustments to achieve more successful outcomes. Communications between providers and parents increase the likelihood for effective interventions as children continue to experience communicable or noncommunicable illnesses into later childhood. Unrecognized gene–environment interactions during the first 1,000 days later contribute to the child’s resilience or vulnerability as neurologic and mental health or disease is expressed at older ages (9).

Healthy pregnancies followed by uneventful births consequently will receive less provider surveillance and will be a greater challenge for the trainee. This asymptomatic majority (26) nonetheless may later develop sequelae after the first 1,000 days. Many children may still overcome identified risks, exhibiting only transient abnormal clinical signs with functional neurodivergence requiring fewer interventions at older ages. This mainstreamed cohort alternatively may later experience adolescent neurologic and mental health disorders. Long prodromal periods after prenatal disease onset often precede later childhood neurologic and mental health phenotypic presentations. Brain health care combined with educational bundles more effectively offer interventions for children identified by using a proposed IFNNC database. Documented reproductive, prenatal and preschool risk factors can be applied to educational plans throughout the school years despite minimal or no clinical expressions before school entry. Strategies by teachers and health providers help maintain critical thinking to provide equitable services for all children, including those challenged by neurologic and psychiatric disorders (104). Educational plans will more likely remain effective even when problematic neurodivergence is identified. Co-morbid mental health disorders continue to have life-changing negative consequences through adolescence given bidirectional negative interactions with neurologic disorders. The trainee’s knowledge of interventions during school years into early adulthood can better appreciate benefits to a greater number of young adults who will seek college education or vocational training to secure employment with independent living.

## Connection of IFNNC knowledge silos are strengthened by adult neuroscience disciplines

Activities of the IFNNC emphasize for trainees continuity of brain care bundles that help sustain brain health into adulthood (105). IFNNC certification endorsed by adult neurology and mental health professional organizations can be modified for adult providers to more effectively provide healthcare for adults previously identified as children with neurologic and mental health disorders. A broad range of neurologic subspecialties would benefit from discipline-specific certifications to better identify and treat adult brain disorders by considering developmental origins that were identified earlier in the life cycle. IFNNC competencies will help adult neurologic and mental health providers in communities particularly where resource challenges exist. International cooperation among organizations offers public health and economic policies promoted by the World Health Organization and the World Economic Forum, to provide adult brain health care based on information acquired by partnering with the IFNNC. Adult brain health care providers can more effectively advocate for preventive strategies based on recommended lifestyle interventions (106) that will later strengthen neurorescue and neuroreparative strategies for brain disorders such as stroke and dementias experienced into senescence.

**Figure 2:**
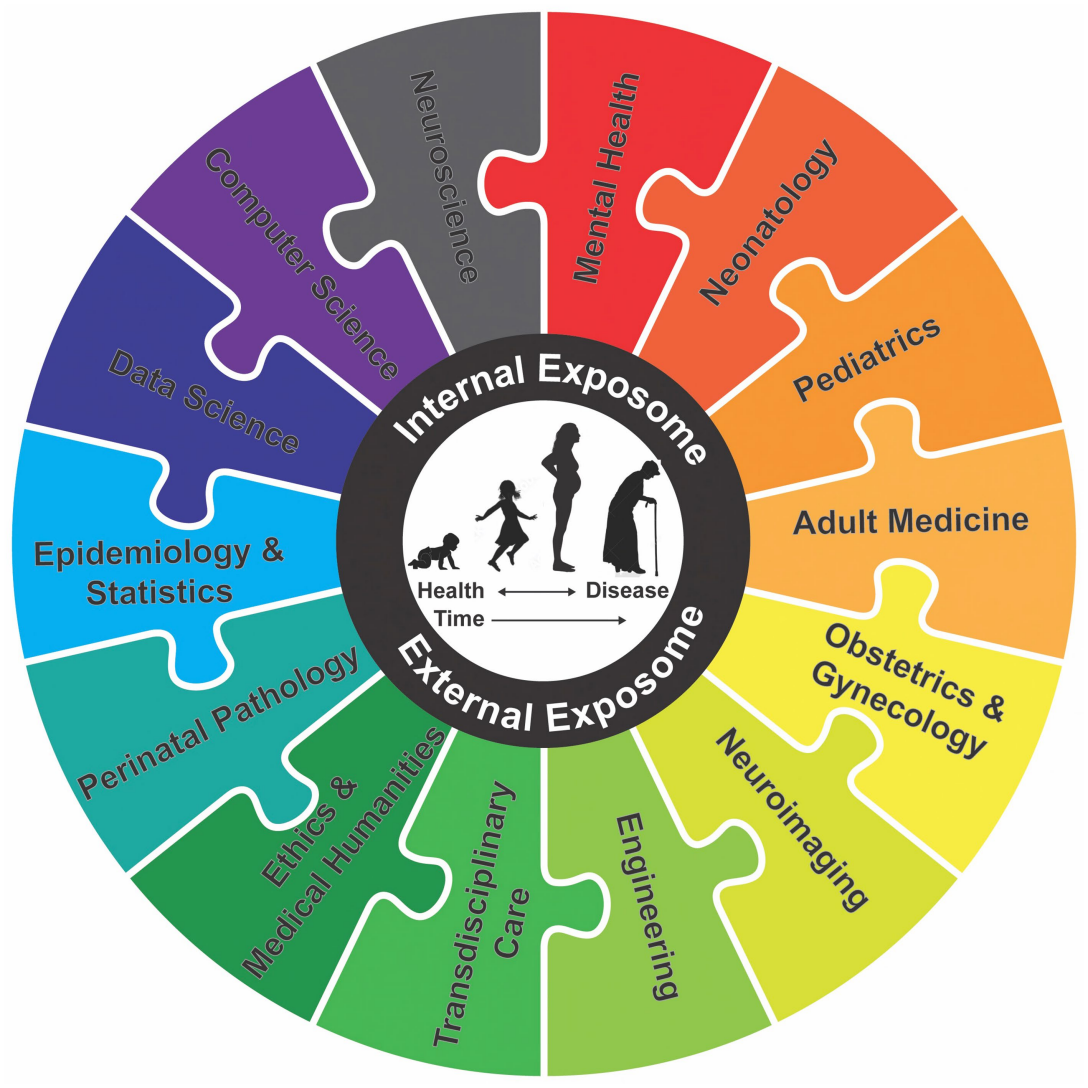
Interlocking puzzle pieces represent synergy among three integrated clusters of a proposed IFNNC. Time-sensitive approaches will enhance life-course brain health. Knowledge of toxic stressor interplay considers combined effects of the internal exposome comprised of biological factors with the external exposome represented by environmental factors. Primary and subspecialty providers partnering with parents include physicians, nurses, midwives, therapists, doulas, social workers, child-life specialists and family advocates. Brain care bundles adjust to the child’s health and disease initially over the first 1,000 days with collective input among disciplines. Perinatal pathologists for example benefit from consultation with neuropathology and neuroembryology colleagues. Engineers and computer science experts offer artificial intelligence with machine learning to assist epidemiologic and statistical investigations for research and public health policy efforts. Shared decisions with patients and families introduce humanistic and ethics-based values through storytelling applied to transdisciplinary care with agency. Transition after formal education with career-long experiences by providers strengthen neurologic and mental health outcomes. Adult neurology and mental health provider membership with a proposed IFNNC maintain important developmental origins of health and disease perspectives during each person’s workplace experiences through retirement and senescence. Diagnostic and neurotherapeutic choices across the lifecycle benefit from this proposed IFNNC approach for life-course brain health [modified from (111, 128)].

Life cycle collaborative efforts must begin with planning during the first 1,000 days. Maternal and pediatric-siloed disciplines however remain predominately isolated, including obstetrics, maternal-fetal medicine (67, 107), neonatology (108), perinatal pathology (109), midwifery (110), neuroradiology (111), clinical neurophysiology (112), psychology, genetics (100, 113) and computational science (114). Connecting these siloes by the proposed IFNNC early during the life cycle will generate useful data for adult neurology and mental health providers. All stakeholders will be encouraged to participate in a lifelong brain health information exchange platform that offers interventions from development through aging (115) (Figure 2).

Fetal and neonatal developments in biomarker research will improve identification of vulnerable pediatric and adult populations for neuroprotective interventions. Comparisons of prenatal with postnatal brain structure and function will better recognize asymptomatic as well as symptomatic populations. Serial studies can help track time-sensitive disease pathways that often remain unrecognized for years or decades before older child or adult phenotypes appear. Placental exome analyses during the woman’s pregnancy for example can monitor trophoblastic functions that correlate with healthy or compromised fetal brain health when compared with other organ systems. Comparisons of these findings with serial magnetic resonance imaging of the maternal-placental-fetal triad, neonate and child will beter document expected or aberrant neuronal maturational trajectories. Such diagnostic data will help formulate therapeutic interventions initially during the first 1,000 days that will improve outcomes (116). Later childhood and adult brain health strategies can be designed also using IFNNC-generated data to sustain wellness and treat disease expressions across the lifespan.

The IFNNC board of directors as well as steering, membership, scientific and education committees would benefit from stakeholder representatives who advocate for life-course brain health. Adult provider participation offers “developmental origins of health and disease” perspectives for all collaborative participants by applying IFNNC-generated data. Diagnostic strategies can more effectively be designed to monitor neurological performances with interventions across the lifespan. Preventive neuroprotective choices during early adulthood promote healthy nutrition, sleep, exercise and mental health. These wellness measures help reduce the later occurrence and severity of neurological disorders expressed by older adults, such as Alzheimer’s and related dementias (117). More effective interventions for neurodegenerative, cerebrovascular and mental health diseases can also be designed based on life-course IFNNC-generated information that considered early life cycle experiences as later life interventions are offered. Knowledge of women’s and children’s health will help strengthen the selection of adult neurology and mental health care interventions (9) based on life-course and developmental origins concepts.

Large scale global diplomacy can successfully achieve greater equity in brain health public policy (118), integrating preventive, rescue and reparative interventions during each part of the life cycle. Peer-review journals with editorial endorsements currently promote commissions, emphasizing intersectionality to advocate for equitable public health policies using systems-science. These same priorities needed to be explained in standard multi-authored medical texts. Regularly scheduled international summits such as in Davos Switzerland principally represented by the G7 nations provide opportunities for an exchange of ideas that contribute brain health strategies in resource adequate and deprived communities. Media platforms can disseminate scientific and socially responsible information through webinars, podcasts and chat rooms to encourage dialog and public health efforts. The proposed IFNNC must advocate training that first considers women and children when promoting life cycle health care efforts.

University hubs comprised of multiple schools and departments contribute to this proposed IFNNC (119). Women’s health, pediatric and adult neurology, and mental health expertise at these hubs can collaborate with physical and social sciences, humanities, legal-ethics and public policy faculty to offer more comprehensive approaches to life-course brain health. Lack of specific health resources may exist at any one institution such as maternal, placental, and fetal brain MRI imaging, neonatal neurophysiologic testing, or perinatal pathology studies. These centers alternatively can invest in engineering and computer science expertise to accurately record and process data using artificial intelligence and machine learning methodologies. Completion of data processing at designated academic hubs can subsequently create information to be used for patient care, education, and research purposes relevant to the specific communities that are served. Academic hubs with a more complete complement of resources more effectively can participate in multi-institutional research activities, supported by competitive grants, philanthropic and industry support.

Decision-making algorithms must minimize errors of omission and commission by using critical thinking based on epidemiologic and statistical oversight tools (120). Longitudinal non-randomized intervention protocols using acyclic directed graphs (121) select appropriately powered population stratification that combines relevant demographic and clinical factors that recognize intersectionality effects on a dynamic neural exposome. Randomized controlled trials can subsequently be designed to assess more narrowly defined cohorts. These will be based on hypotheses from previously described longitudinal cohorts that applied causal inference theory to assess brain health or disease. Life-course research protocols will assess adaptive or maladaptive neuroplasticity by considering reproductive, pregnancy and pediatric exposome influences beginning before the first 1,000 days (9). Life-course brain health effects consequently can more accurately assess intragenerational and transgenerational outcomes (122).

Interdisciplinary didactic course curricula offer classroom and online instruction for IFNNC participants. Electronic libraries with webinar-based lectures and small group discussions enhance learning experiences. Professional societies will align their certification and board examination requirements to offer onsite or remote courses with invaluable educational services inclusive of trainees and faculty in resource-challenged communities. Lack of funds, no internet access, regional armed conflicts, geopolitical unrest, climate changes and the paucity of the full complement of health providers in more remote and vulnerable areas can more effectively be addressed through international cooperation that provides equitable training, practice and research opportunities. These efforts will identify future leaders who will continue to promote interdisciplinary collaborations through improved public health programs that advocate for global neurologic and mental health.

## Advocacy groups raise public awareness to support IFNNC activities

Shared decisions among multiple providers and parents build trust and maintain engagement to raise public awareness through responsible communication platforms and social media. Such efforts help increase public support, legislative reform and funding opportunities through advocacy with those who share their stories as women, children and families based on their lived experiences. Activities by non-governmental organizations exemplify these efforts such as the “Every Women-Every Child” WHO program (123) and the United States-based “Healthy Moms-Strong Babies” project launched by the March of Dimes (44). Involvement by parent advocacy organizations such as Neopedia, Hope for HIE, Every Mother Counts, and United We Push are examples of important stakeholders to be recognized by trainees. Participation by parents and families who have experienced fetal, neonatal and childhood brain disorders (124) greatly enrich IFNNC activities by sharing their personal memories through effective communication. Multimedia art offers brain health experiences that also benefit providers. These neurohumanistic approaches enhance practice, educational and research objectives by highlighting a person’s lifecycle experiences from childhood into adulthood.

## Preventive neurology and psychiatry programs help promote worldwide brain health

This proposed two-year IFNNC supports more comprehensive formal training than previously suggested based on one-year programs. Trainees will consequentially be better prepared to increase their expertise with career-long experiences. Increased funding will be a challenge given this longer training period and will need to rely on increased institutional, governmental or philanthropic support. Trainees will also experience lower income during a longer training period. However, more extensive training will better prepare future providers to consider neuroprotective options as scientific and public health advances are developed and modified (1, 9). Proactive preventive neurology and mental health strategies should continue to be stressed beginning before each pregnancy. These early interventions promote interdisciplinary diagnostic and interventions to improve the outcomes for MPF triads, neonates and children. Empowering women to participate in healthcare for themselves and their children will remain paramount in importance. A developmental origins of health and disease approach adopted by these collaborative programs will connect preconception and childhood with adult life experiences, emphasizing intersectionality to achieve equitable brain health across the lifespan (125). This proposed IFNNC training will augment providers in general practice, neurology and mental health by participation in life-course integrative brain health care. Future curriculum revisions based on scientific advances offer opportunities for improved interdisciplinary training (18). Shared knowledge among disciplines reduces the gap with practice delivery by enhancing neuroscience educational goals that support research efforts to design of more effective interventions (126).

Life course brain health strategies (21) will more effectively address the worldwide public health crisis involving over one third of the world’s population who experience neurologic and mental disorders across the lifespan (2, 125, 127). Pediatric neurologic disorders significantly contribute to this global burden of brain disorders. Earlier identification regarding challenges to girls’ and women’s health care can positively influence brain health across the life cycle. Providers throughout the Global South must preferentially rely on preventive brain health measures to promote childhood and women’s healthcare as their nations invest in more sophisticated medical centers. These proactive measures will nonetheless strengthen rescue and reparative neuroprotective choices as more advanced interventions are made available. Resource limitations, geographical isolation and ethical-cultural-religious differences will continue to remain challenges to overcome. Transdisciplinary healthcare practices by all stakeholders will help advocate for the continuity of brain care bundles with benefits over successive generations. Participation by those with lived experiences through storytelling using multi-media art will strengthen clinical decisions by recognizing neurohumanism (13). These proposed IFNNC educational, practice and research activities will offer a more complete tool chest of complementary neuroprotective strategies to achieve wellbeing, brain health and an improved quality of life.

## Footnotes

1
www.IHI.org

2
www.UCNS.org
