# Supplementary material for: An interdisciplinary fetal neonatal neurology collaborative promotes integrative life-course brain health
Source: Front Neurol. 2026 Jan 7;16:1725289. doi: 10.3389/fneur.2025.1725289 (PMC12819184; doi:10.3389/fneur.2025.1725289)
Supplement: Supplementary file 1 [file Presentation_1.pptx]

## Slide 1
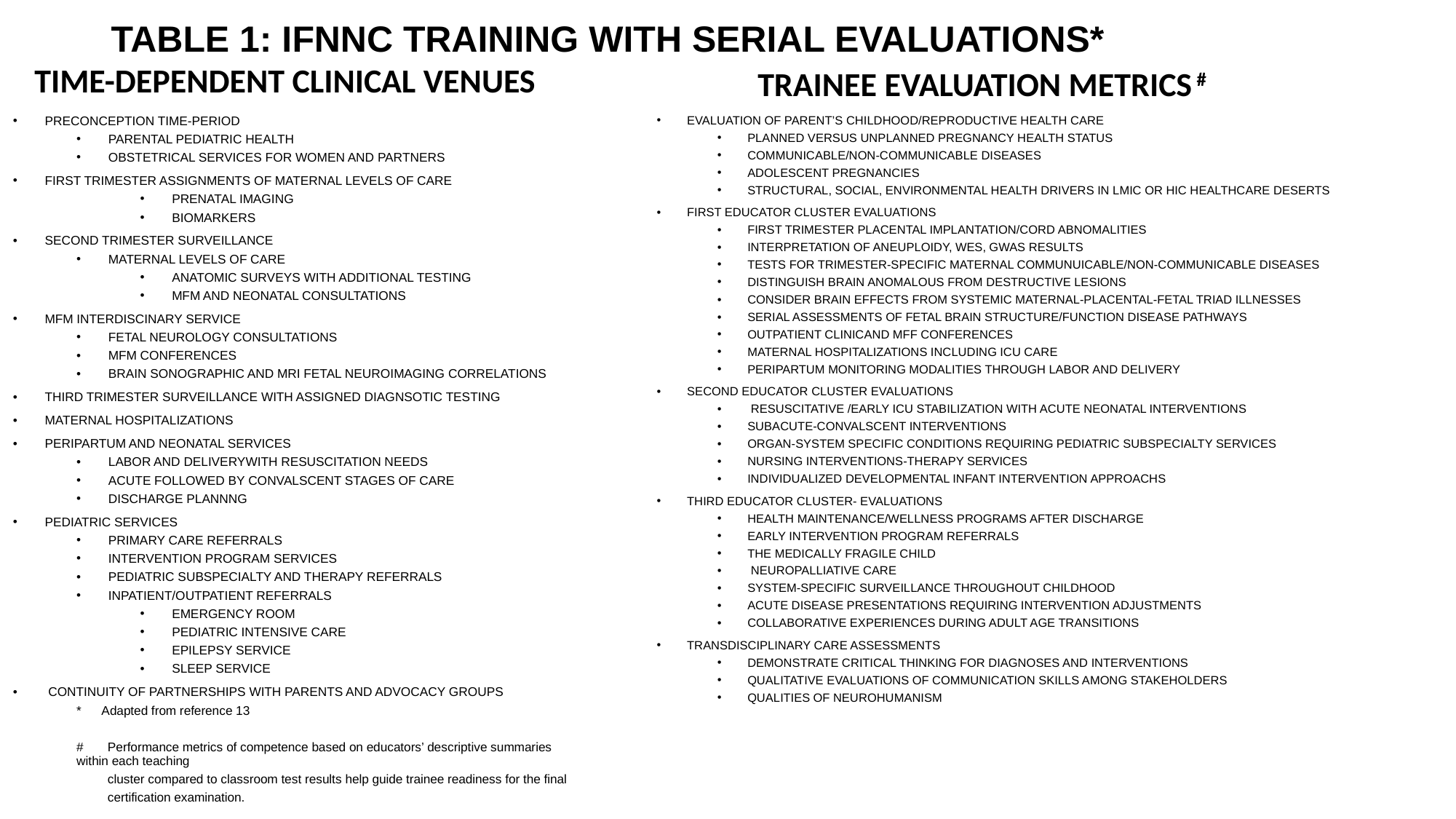

# TABLE 1: IFNNC TRAINING WITH SERIAL EVALUATIONS*
 TIME-DEPENDENT CLINICAL VENUES
 TRAINEE EVALUATION METRICS #
PRECONCEPTION TIME-PERIOD
PARENTAL PEDIATRIC HEALTH
OBSTETRICAL SERVICES FOR WOMEN AND PARTNERS
FIRST TRIMESTER ASSIGNMENTS OF MATERNAL LEVELS OF CARE
PRENATAL IMAGING
BIOMARKERS
SECOND TRIMESTER SURVEILLANCE
MATERNAL LEVELS OF CARE
ANATOMIC SURVEYS WITH ADDITIONAL TESTING
MFM AND NEONATAL CONSULTATIONS
MFM INTERDISCINARY SERVICE
FETAL NEUROLOGY CONSULTATIONS
MFM CONFERENCES
BRAIN SONOGRAPHIC AND MRI FETAL NEUROIMAGING CORRELATIONS
THIRD TRIMESTER SURVEILLANCE WITH ASSIGNED DIAGNSOTIC TESTING
MATERNAL HOSPITALIZATIONS
PERIPARTUM AND NEONATAL SERVICES
LABOR AND DELIVERYWITH RESUSCITATION NEEDS
ACUTE FOLLOWED BY CONVALSCENT STAGES OF CARE
DISCHARGE PLANNNG
PEDIATRIC SERVICES
PRIMARY CARE REFERRALS
INTERVENTION PROGRAM SERVICES
PEDIATRIC SUBSPECIALTY AND THERAPY REFERRALS
INPATIENT/OUTPATIENT REFERRALS
EMERGENCY ROOM
PEDIATRIC INTENSIVE CARE
EPILEPSY SERVICE
SLEEP SERVICE
 CONTINUITY OF PARTNERSHIPS WITH PARENTS AND ADVOCACY GROUPS
* Adapted from reference 13
# Performance metrics of competence based on educators’ descriptive summaries within each teaching
 cluster compared to classroom test results help guide trainee readiness for the final
 certification examination.
EVALUATION OF PARENT’S CHILDHOOD/REPRODUCTIVE HEALTH CARE
PLANNED VERSUS UNPLANNED PREGNANCY HEALTH STATUS
COMMUNICABLE/NON-COMMUNICABLE DISEASES
ADOLESCENT PREGNANCIES
STRUCTURAL, SOCIAL, ENVIRONMENTAL HEALTH DRIVERS IN LMIC OR HIC HEALTHCARE DESERTS
FIRST EDUCATOR CLUSTER EVALUATIONS
FIRST TRIMESTER PLACENTAL IMPLANTATION/CORD ABNOMALITIES
INTERPRETATION OF ANEUPLOIDY, WES, GWAS RESULTS
TESTS FOR TRIMESTER-SPECIFIC MATERNAL COMMUNUICABLE/NON-COMMUNICABLE DISEASES
DISTINGUISH BRAIN ANOMALOUS FROM DESTRUCTIVE LESIONS
CONSIDER BRAIN EFFECTS FROM SYSTEMIC MATERNAL-PLACENTAL-FETAL TRIAD ILLNESSES
SERIAL ASSESSMENTS OF FETAL BRAIN STRUCTURE/FUNCTION DISEASE PATHWAYS
OUTPATIENT CLINICAND MFF CONFERENCES
MATERNAL HOSPITALIZATIONS INCLUDING ICU CARE
PERIPARTUM MONITORING MODALITIES THROUGH LABOR AND DELIVERY
SECOND EDUCATOR CLUSTER EVALUATIONS
 RESUSCITATIVE /EARLY ICU STABILIZATION WITH ACUTE NEONATAL INTERVENTIONS
SUBACUTE-CONVALSCENT INTERVENTIONS
ORGAN-SYSTEM SPECIFIC CONDITIONS REQUIRING PEDIATRIC SUBSPECIALTY SERVICES
NURSING INTERVENTIONS-THERAPY SERVICES
INDIVIDUALIZED DEVELOPMENTAL INFANT INTERVENTION APPROACHS
THIRD EDUCATOR CLUSTER- EVALUATIONS
HEALTH MAINTENANCE/WELLNESS PROGRAMS AFTER DISCHARGE
EARLY INTERVENTION PROGRAM REFERRALS
THE MEDICALLY FRAGILE CHILD
 NEUROPALLIATIVE CARE
SYSTEM-SPECIFIC SURVEILLANCE THROUGHOUT CHILDHOOD
ACUTE DISEASE PRESENTATIONS REQUIRING INTERVENTION ADJUSTMENTS
COLLABORATIVE EXPERIENCES DURING ADULT AGE TRANSITIONS
TRANSDISCIPLINARY CARE ASSESSMENTS
DEMONSTRATE CRITICAL THINKING FOR DIAGNOSES AND INTERVENTIONS
QUALITATIVE EVALUATIONS OF COMMUNICATION SKILLS AMONG STAKEHOLDERS
QUALITIES OF NEUROHUMANISM
